# Role of Tactile Noise in the Control of Digit Normal Force

**DOI:** 10.3389/fpsyg.2021.612558

**Published:** 2021-02-12

**Authors:** Abdeldjallil Naceri, Yasemin B. Gultekin, Alessandro Moscatelli, Marc O. Ernst

**Affiliations:** ^1^Advanced Robotics Department, Italian Institute of Technology, Genoa, Italy; ^2^Neurobiology of Vocal Communication, Werner Reichardt Centre for Integrative Neuroscience, University of Tübingen, Tübingen, Germany; ^3^Functional Imaging Laboratory, German Primate Center, Leibniz Institute for Primate Research, Göttingen, Germany; ^4^Department of Systems Medicine and Centre of Space Biomedicine, University of Rome Tor Vergata, Rome, Italy; ^5^Laboratory of Neuromotor Physiology, Istituto di Ricovero e Cura a Carattere Scientifico (IRCCS) Fondazione Santa Lucia, Rome, Italy; ^6^Applied Cognitive Psychology, Faculty for Computer Science, Engineering, and Psychology, Ulm University, Ulm, Germany

**Keywords:** grasping, manipulation, tactile perturbation, normal force, tactile object

## Abstract

Whenever we grasp and lift an object, our tactile system provides important information on the contact location and the force exerted on our skin. The human brain integrates signals from multiple sites for a coherent representation of object shape, inertia, weight, and other material properties. It is still an open question whether the control of grasp force occurs at the level of individual fingers or whether it is also influenced by the control and the signals from the other fingers of the same hand. In this work, we approached this question by asking participants to lift, transport, and replace a sensorized object, using three- and four-digit grasp. Tactile input was altered by covering participant's fingertips with a rubber thimble, which reduced the reliability of the tactile sensory input. In different experimental conditions, we covered between one and three fingers opposing the thumb. Normal forces at each finger and the thumb were recorded while grasping and holding the object, with and without the thimble. Consistently with previous studies, reducing tactile sensitivity increased the overall grasping force. The gasping force increased in the covered finger, whereas it did not change from baseline in the remaining bare fingers (except the thumb for equilibrium constraints). Digit placement and object tilt were not systematically affected by rubber thimble conditions. Our results suggest that, in each finger opposing thumb, digit normal force is controlled locally in response to the applied tactile perturbation.

## Introduction

It is possible to grasp the same object in an infinite number of ways, due to the multiple combinations of digit placement on the object and the level of force exerted by each digit. Grasping and manipulating an object requires that the central nervous system masters this redundancy in degrees of freedom (Bernshtein, 1967; Naceri et al., 2014, 2017). Sensory feedback from proprioception, touch, and other modalities such as vision plays a key role in the control of grasping. In particular, tactile feedback is conveyed by afferent fibers that respond to the deformation of the mechanoreceptors in the skin. Cutaneous mechanoreceptors provide information concerning the timing of the contact, the location of the contact area on the skin, and the direction and amplitude of the contact force (Johansson and Flanagan, 2008). In addition to sensory feedback, finger force control is achieved in an anticipatory fashion during grasping tasks, by taking into account prior knowledge of the object and the internalized model of our body (Johansson and Westling, 1984; Westling and Johansson, 1984; Johansson and Flanagan, 2008).

A crucial point for understanding grasping is to uncover the coordination of the different digits, and the integration of cutaneous information from each of them, for motor control. Edin et al. (1992) investigated how the precision grip is regulated with respect to the individual digits. The authors studied precise grasping (two-finger grasping using the thumb and index fingers) while varying the friction coefficient independently at each digit, by changing the contact surfaces. The digit touching the most slippery surface exerted less tangential force than the digit touching the surface with the high friction. Consequently, the safety margins were similar at the two digits. During digital nerve block, large, and variable safety margins were employed, i.e., in absence of feedback, the finger-tip forces were not related to surface properties. Burstedt et al. (1999) extend the previous study to tripod grasping, i.e., three-digit grasping using index, middle finger, and thumb. The two studies reported that digit normal forces were adjusted locally when changing the frictional conditions at fingertip contact. Aoki et al. (2007) investigated the same research question for five-digit grasps using a similar experimental method and paradigm. Specifically, they manipulated the digit contact friction using either low friction (rayon paper) or high friction (sandpaper) independently for each digit giving 32 possible combinations (Aoki et al., 2007). They observed nonlocal force changes (between digit control), when the friction coefficient was changed in at least three digits.

A fourth study, which used a similar paradigm as the aforementioned study, revealed a similar within and between digit normal force regulation during five-digit grasps (McIsaac et al., 2009). This latter study concluded that multidigit force responses to texture, which was revealed by the studies referenced earlier, are not obligatory and instead suggested that the behavioral context of a task ought to be considered when inferring general principles of multidigit force coordination.

As indicated above, the nature of force control during grasping tasks is still unclear. The referenced studies investigated the modulation of a precision grip under different frictional conditions at the fingertips. All these studies used a manipulandum that constrained digit location due to the fixed location of force transducers. However, digit locations play a major role in influencing the modulation of the digit forces (Burstedt et al., 1999; Fu et al., 2010; Naceri et al., 2014).

In this study, the tactile input to different fingertips was perturbed by asking participants to wear a rubber thimble, which reduced the reliability of the tactile signal and changed the friction coefficient between finger and object (Kinoshita, 1999). First, we investigated whether the normal force exerted by each digit varies when wearing a rubber thimble. Additionally, we evaluated whether the thimble modulates the contact force locally, i.e., the force exerted by the covered finger, or globally, when grasping with three and four digits. We exploited the technical advantages of an innovative sensorized object to study the above question during unconstrained hand grasping (Naceri et al., 2017). In each trial, participants were required to grasp, lift, transport, and replace the sensorized object. Due to the design of the sensorized object, in each trial, participants could freely choose the location of the digits on the object. We considered two possible motor strategies, where force is controlled either independently within each digit or simultaneously across digits. In the former, we expect that wearing the thimble might increase or decrease grip force without affecting the adjacent noncovered finger. In the across digit normal-force regulation, we expect a synergistic increase or decrease across the adjacent noncovered fingers. The thumbs' normal force should be equal to the sum of normal forces of the opposing fingers in order to achieve a stable grasp. We hypothesize that in three-digit grasps, there is a within-digit control. Moreover, for four-digit grasping, synergic effects may occur between fingers (Santello et al., 2013). Therefore, in four-digit grasps, we expect between-digit force control rather than within-digit force control.

## Method

### Participants

Twenty-eight right-handed participants took part in the experiment (13 females, 15 males; age 27 ± 5 years of age, and mean ± standard deviation). Participants received course credit or were paid 7 €/h for their participation. Ten out of 28 participants participated in more than one condition with the thimble covering a different number of fingers; at least 48 h passed between two conditions. Six participants participated in all conditions. All participants had no history of neurological or motor deficits. The testing procedures were approved by the ethics committee of Bielefeld University and were conducted in accordance with the guidelines of the Declaration of Helsinki for research involving human participants. Informed written consent was obtained from all participants involved.

### Experimental Setup

With the TACtile-sensorized Object (TACO), we are able to record the position and the normal force exerted by each finger on its sensorized surface, while it allows participants to freely choose the position for digit placement on the object, thus enabling unconstrained grasping (Schürmann et al., 2011). For details on using this device for gasping studies, see also Naceri et al. (2017). For this study, an additional force/torque sensor was integrated in the center of the TACO to additionally record the net torque and force vectors (see below). The TACO has a cuboid shape [length (*l*) = 170 mm; height (*h*) = 85 mm; width (*w*) = 55 mm], while the two opposing sides used for grasping were sensorized with four high-speed tactile sensor modules (Schürmann et al., 2011), named “Myrmex”—two on each side (Schürmann et al., 2012). Each Myrmex module provides a 16 × 16 tactile sensor array on an area of 80 × 80 mm^2^, has a sampling rate of up to 1.9 kHz, and a spatial resolution of 5 mm (the size of one sensor element). That is, the TACO consisted of 512 tactels on each side. The Myrmex sensors were covered with conductive foam in order to increases the dynamic range of the sensor to match the contact pressure during grasping (Schürmann et al., 2012). The TACO allows us to simultaneously record the center of pressure and the normal force exerted by each digit. The device was calibrated using a force gauge with a force ranging from 0 to 25 N. During the calibration, we varied the cross-sectional area of the gauge tip from 10 to 50 mm^2^ with a step of 20 mm^2^ to account for the differences in size of participants' fingertips. The six-axis force/torque sensor that was added in the center of the TACO was the “Mini40” from ATI.

Two metallic plates were fixed on both sides of the force/torque sensor through its threaded insert. Each metallic plate was then attached to one side of the TACO using four screws. The force/torque sensor allowed us to record the net forces and net torques of participants' grasp.

**Figure 1:**
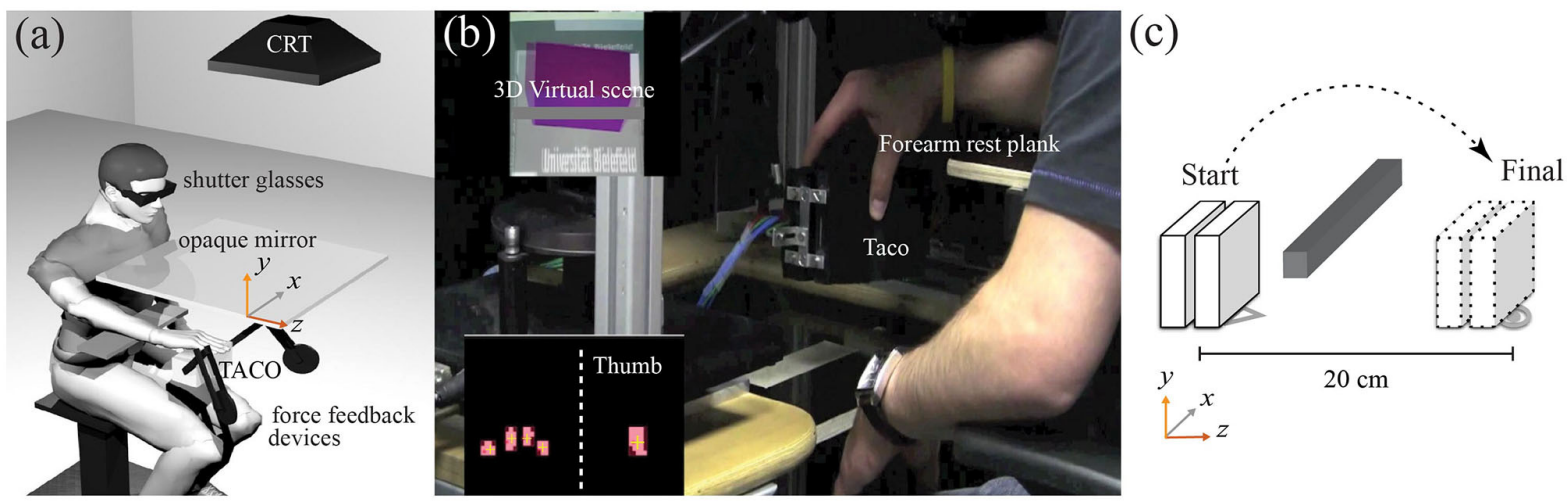
Experimental setup. **(a)** Participants binocularly viewing the mirror image of the visual scene. **(b)** The TACO attached to the PHANToMTM force feedback devices. On the top left, the 3D virtual scene, the purple cube color indicates to the participants that the TACO collided the gray horizontal bar (red color otherwise). On the bottom left, the TACO output image with a yellow cross represents digit center of pressures (CoPs). **(c)** The TACO initial and final target and its desired trajectory.

During the experiment, participants looked through a mirror onto the haptic scene. The mirror occluded the participants' hands and the TACO from sight thus removing any visual feedback about their hand location and grasp. The mirror faced a computer monitor (21-in. CRT-computer monitor SONY CPDG520 with a resolution of 1280–1024 pixels and refresh rate of 100 Hz) that we used to display the visual stimulus. The visual stimulus consists of a rectangular cuboid, with the same dimensions of the TACO. Participants wore liquid-crystal shutter glasses (CrystalEyes™) providing binocular disparity (Figure 1a).

The TACO was attached to two PHANToM™ (SensAble Technologies) force-feedback devices to track its position and allow force/torque perturbations to be applied during the holding phase of the trial (Figure 1b). The sampling rate of the PHANToM^™^ was 1 kHz, allowing a precise position match between the virtual visual simulation and the physical TACO object. The total mass of TACO was 0.850 kg. Due to the constraints imposed by the two PHANToM^™^ devices, the TACO had 5 degrees of freedom: translation along the *x, y*, and *z* directions, and rotation along the yaw (***α***) and roll (***δ***) axis. The 3D position and the rotations around the yaw and roll axis of the TACO were tracked using the PHANToM^™^ devices.

### Procedure

Participants sat on a chair with adjustable height. Before the start of the grasping movement, participants received an auditory “GO” signal, instructing them to grasp the TACO and to move it from the start to the end location behind a virtual bar, as illustrated in Figure 1c. They were asked to move the object smoothly and to place it in the end position in a similar orientation as it was picked up back at the beginning of the trial. A simulated rectangular cuboid was displayed on the CRT via the mirror with matched size and in the same location as the TACO. To provide participants with feedback, the virtual cuboid changed its color from red to purple as if it collided with the virtual bar (Figure 1b, upper left panel). After participants placed the TACO on the table, they received a second auditory signal prompting them to release the TACO and the experimenter stopped data recording. The next auditory signal instructed participants to replace the TACO back to the starting position for the next trial (Figure 1c). In different experiments, participants were instructed to grasp the TACO with three or four digits (thumb-index-middle digits, thumb-index-middle-ring digits). Fingers not involved in the task were extended and taped to a splint made of cardboard to prevent them from contacting the TACO. The finger placements on the TACO were self-chosen (grasping without constraints). There were different grasping conditions depending on how many fingers opposing the thumb were covered with a rubber thimble made from three layers of a nitrile glove, starting from none (control condition), to one, two, or three. Each trial lasted ~10 s from grasp onset to the end. Fourteen trials were conducted for each condition. Each experimental session consisted of one condition involving the glove together with the baseline condition with no finger covered by a glove. The order of the experimental and control condition was randomly assigned to the participants. The total duration of the experimental session was approximately 15 min. There were two grasp conditions (three-digit grasp and four-digit grasp) and thus giving four glove conditions for the three-digit grasps: (1) no glove, (2) index finger covered, (3) middle finger covered, and (4) index and middle fingers covered and eight glove conditions for the four-digit grasps: (1) no glove, (2) index finger, (3) covered, (4) middle finger covered, (4) ring finger covered, (5) index and middle fingers covered, (6) index and ring fingers covered, (7) middle and ring fingers covered, and (8) index, middle, and ring fingers covered.

### Data Processing and Analysis

**Figure 2:**
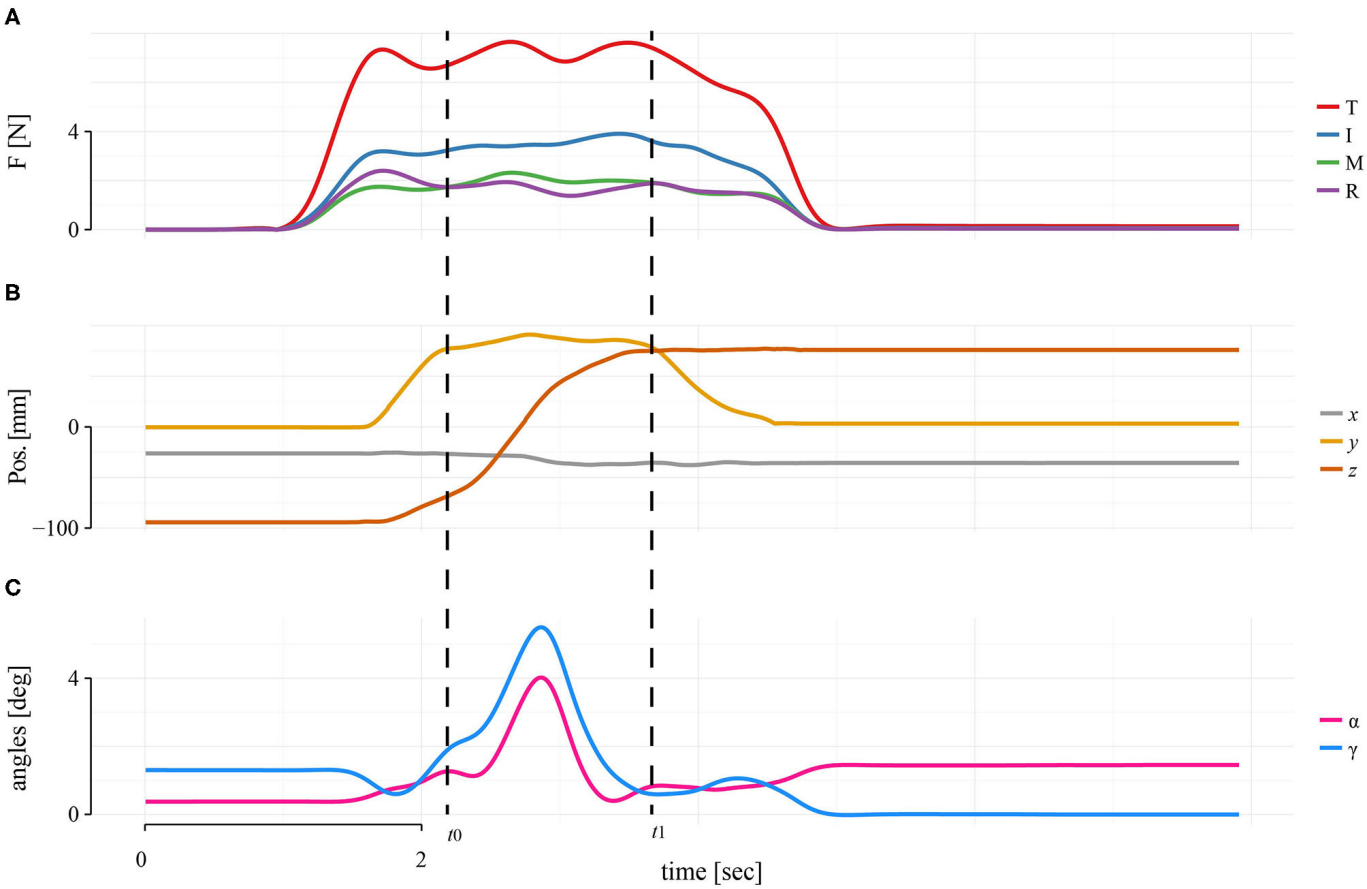
Single trial of a representative participant in four digits in baseline condition. **(A)** Force profiles for each digit. **(B)** TACO coordinates. **(C)** TACO orientations. Dashed lines represent the transporting phase, which is the interval between *t*_0_ and *t*_1_, where all dependent variables were quantified and averaged.

Using the TACO device, we measured the values of normal force (*F*) for each finger and their positions on the surface of the device, determined by the horizontal (CoP_*x*_) and vertical center of pressures (CoP_*y*_) independently for each finger. We set the center of the TACO as the origin of the coordinate system (0, 0, 0). The CoP_*x*_ and CoP_*y*_ were defined as the location of the global maximum of the activated region of tactels for each fingers' region on the sensor matrix. The force measurements were converted from arbitrary units to Newtons using the lookup table generated during calibration. The calibration table was obtained with a resolution of ± 0.2 N. Digit locations, normal forces, and the position and orientation of the TACO were recorded and processed with a second-order Butterworth low-pass filter with 10 Hz cutoff frequency (Figure 2). In the following, we analyzed the average values of CoP_*x*_ and CoP_*y*_ during the transport phase of the TACO (i.e., the CoP values recorded between time *t*_0_ and time *t*_1_ in Figure 2).

For each digit contact with its CoP, we determined the force vector *F* = (*F*_*x*_, *F*_*y*_, *F*_*z*_) giving the direction and magnitude of the contact force. In the TACO reference frame (Figure 4B), *F*_*z*_ is the normal force *F*^*n*^, *F*_*x*_, and *F*_*y*_ are the tangential force components: *F*^*t*^ = (*F*_*x*_^2^ + *F*_*y*_^2^)^1/2^, that are parallel to the TACO's contact surface.

To satisfy a stable grasp of the TACO, the force produced by the thumb must be equal to the sum of the normal forces of the opposing digits:

(1) $$\sum_{i=T,\;I,\;M,\;R,\;L}F_{i}^{n}=0,\;$$

The sum of the absolute digit normal forces represents the total grip force applied by the participant.

The two PHANToM^™^ devices did not support the weight of TACO. Hence, the sum of digits' load force, $\sum F_{i}^{t}\;$must be equal to the TACO load force when the object is stationary (i.e., when there are no additional inertial forces):

(2) $$\sum_{i=T,\;I,\;M,\;R,\;L}F_{i}^{t}=-\text{mg},\;$$

where *m* is the TACO's mass and *g* is the gravity.

Finally, the friction constraints must be satisfied. Specifically, the force *F* must be inside the friction cone:

(3) $$F_{i}^{t}\;<\;\mu F_{i}^{n},$$

where *μ* is friction coefficient.

### Statistical Analysis

The aim of this study is to evaluate whether normal forces are adjusted either independently for each digit or whether there is a coupling such that the normal forces of some digits are regulated jointly (synergetically), in response to the sensory feedback perturbance when covering individual fingers with a rubber thimble. By means of multivariable linear mixed models (LMMs), we evaluated the systematic variation in the normal force and analyzed whether there are synergetic variations between different digits under the different conditions. The digit normal force “***y***” (**Eq. 4**) was modeled individually for each digit as a linear combination of the experimental variables (referred to as the fixed-effect linear predictors ***Xβ***), the random variability between participants (the random-effect predictors, Zb), and the residual random error *ε* (Bates et al., 2015).

The LMM equation has the following form:

(4) $$y\;=\;\beta_{1}{\text{CoP}}_{\text{x}}\;+\;\beta_{2}\text{G }+\;\beta_{3}{\text{CoP}}_{x}\text{G }+\;\mathrm{Zb}\;+\text{ ε}$$

**Figure 3:**
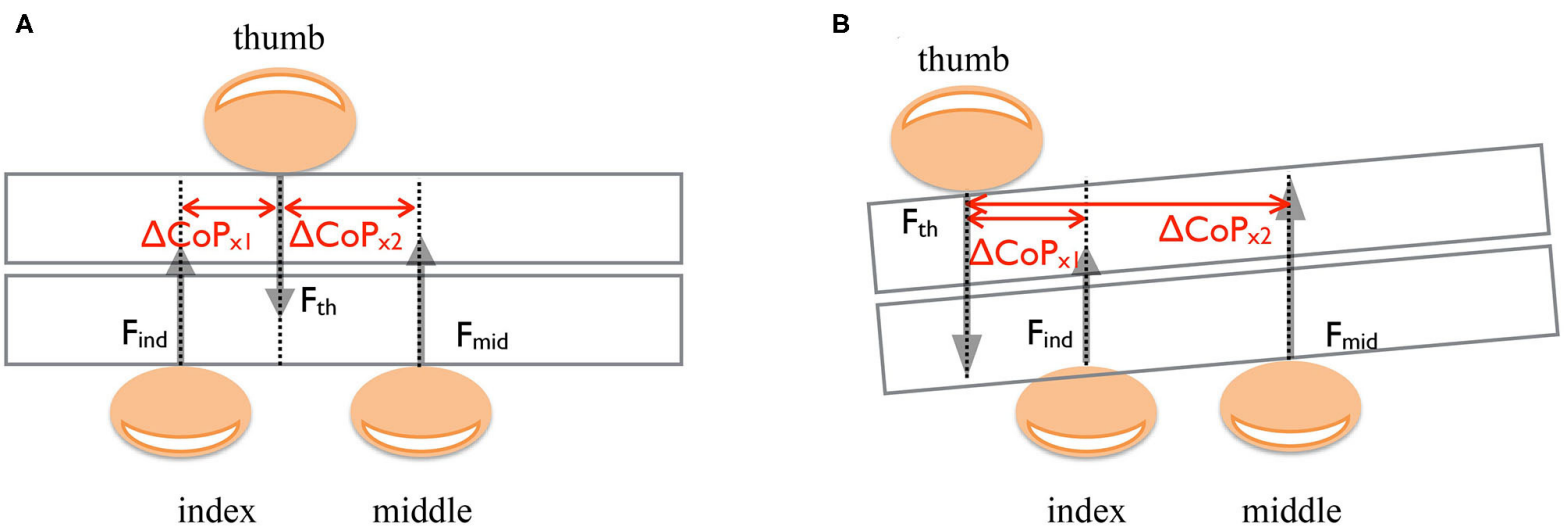
Schema illustrates digit horizontal placements normalized to the thumb, forces, and the TACO orientation in three digits grasp. **(A)** An example of grasp configuration when the thumb is located right between the index and middle fingers. **(B)** Grasp configuration example when the thumb is shifted left of the index and middle fingers.

The matrix of fixed-effect predictors, ***X***, included the following predictors: glove condition *G* (*G* = 1 if any of the fingers was covered with the thimble, and *G* = 0 otherwise), the CoP_*x,y*_ of the index, the middle and the ring fingers normalized to thumb, and the interaction factor between CoP_*x,y*_ and glove condition *G* (Figure 3). We evaluated the effect of each digit by testing the significance of the corresponding single fixed-effect parameters *β*_1_, *β*_2_, and the interaction *β*_3_ with the Likelihood Ratio (LR) test (Pinheiro and Bates, 2000). The LR test compares the maximized log-likelihood functions of two nested models, M1 and M0, with and without the parameter of interest. Under the null hypothesis that the simpler model M0 is better than M1, the LR has a large-sample $\chi_{1}^{2}$ distribution (Bolker et al., 2009; Moscatelli et al., 2012). The dummy variable *G* has value 1 if *at least one* of the fingers was covered with the glove and 0 otherwise. Therefore, the parameter *β*_2_ in **Eq. (4)** allowed us to estimate the influence of the covered finger on the noncovered fingers (see Figure 6).

Next, by using the LMM, we evaluated the effect of the glove perturbation (*G*) and the position CoP of all fingers on the orientation yaw (***α***) and roll (***γ***) of the TACO object:

(5) $$\alpha \;=\;\eta_{1}{\text{CoP}}_{\text{x}}\;+\;\eta_{2}\text{G }+\;\eta_{3}{\text{CoP}}_{\text{x}}\text{G }+\;\mathrm{Zb}\;+\;\text{ε}$$

and

(6) $$\gamma \;=\;\theta_{1}{\text{CoP}}_{\text{x}}\;+\;\theta_{2}\text{G }+\;\theta_{3}{\text{CoP}}_{\text{x}}\text{G }+\;\mathrm{Zb}\;+\;\text{ε}.$$

With this, we could evaluate whether changes of the digit normal forces that were triggered by perturbing the tactile input at each finger would also lead to significant effects on the orientation of the TACO object during the transport phase.

### Predictions

**Figure 4:**
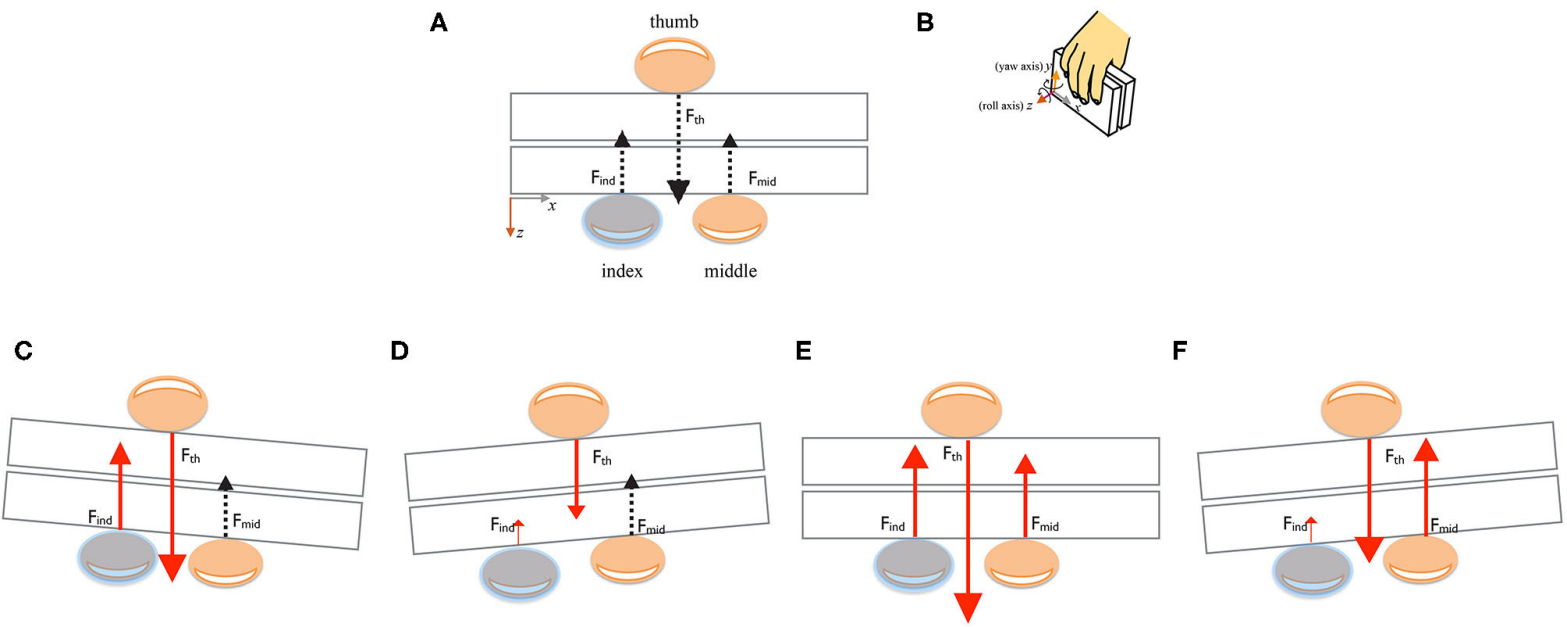
Experimental predictions using three-digit grasp as an example. **(A)** Digit horizontal placements, forces, and TACO position in the baseline condition. **(B)** 3D layout of the human hand grasping the TACO and 3D axes showing the TACO's 5 degrees of freedom. **(C,D)** Predictions for local digit normal force regulation with force increase and decrease of the covered finger, respectively. Black dashed arrow indicates digit normal force of noncovered finger did not change significantly. (**E,F)** Between digit normal force regulations with force increase and decrease at the covered finger, respectively, and the tactile perturbation significantly affecting the noncovered adjacent finger.

In this study, we expect two possible outcomes concerning the control of the digit normal forces: either participants adjusted the grip and normal forces of each digit independently in response to the tactile perturbation (Figures 4C,D), i.e., there is a selective adjustment of normal force in the perturbed finger(s) compared with baseline (Figures 4C,D). Alternatively, there might be a (synergetic) link between the force adjustment of multiple digits even when only some of them are tactilely perturbed (Figures 4E,F), such that the force adjustment occurs across multiple perturbed and nonperturbed fingers. The force variations at single finger can be due to the tactile perturbation from one side and the finger placements with respect to thumb from the other side. In order to exclude (reduce) the effect of force increase due to finger placements with respect to the thumb, we included the distance-normalized CoPs of the fingers to thumb into the LMM (Figure 3).

What complicates the differentiation between these alternative hypotheses is that also under the hypothesis of independent control of finger forces, the nonperturbed fingers might change slightly when the force exerted by the perturbed finger changes in order to meet the stable grasp constraints (**Eq. 1**), i.e., if the force exerted by the perturbed finger decreases, the forces exerted by the other fingers opposing the thumb must increase such that the sum of all normal forces remains zero. For both hypothesis, we may find arguments that the normal force of the perturbed finger(s) might decrease or increase in response to the tactile manipulation. Since the sensitivity of the tactile input is decreased by the rubber thimble, we may speculate that the force might increase to compensate for the reduced input. Alternatively, we may speculate that the unreliable tactile input induced by the rubber thimble might lead to a decrease in force because the feedback for grasp control is less reliable.

## Results

### Three-Digit Grasp

**Figure 5:**
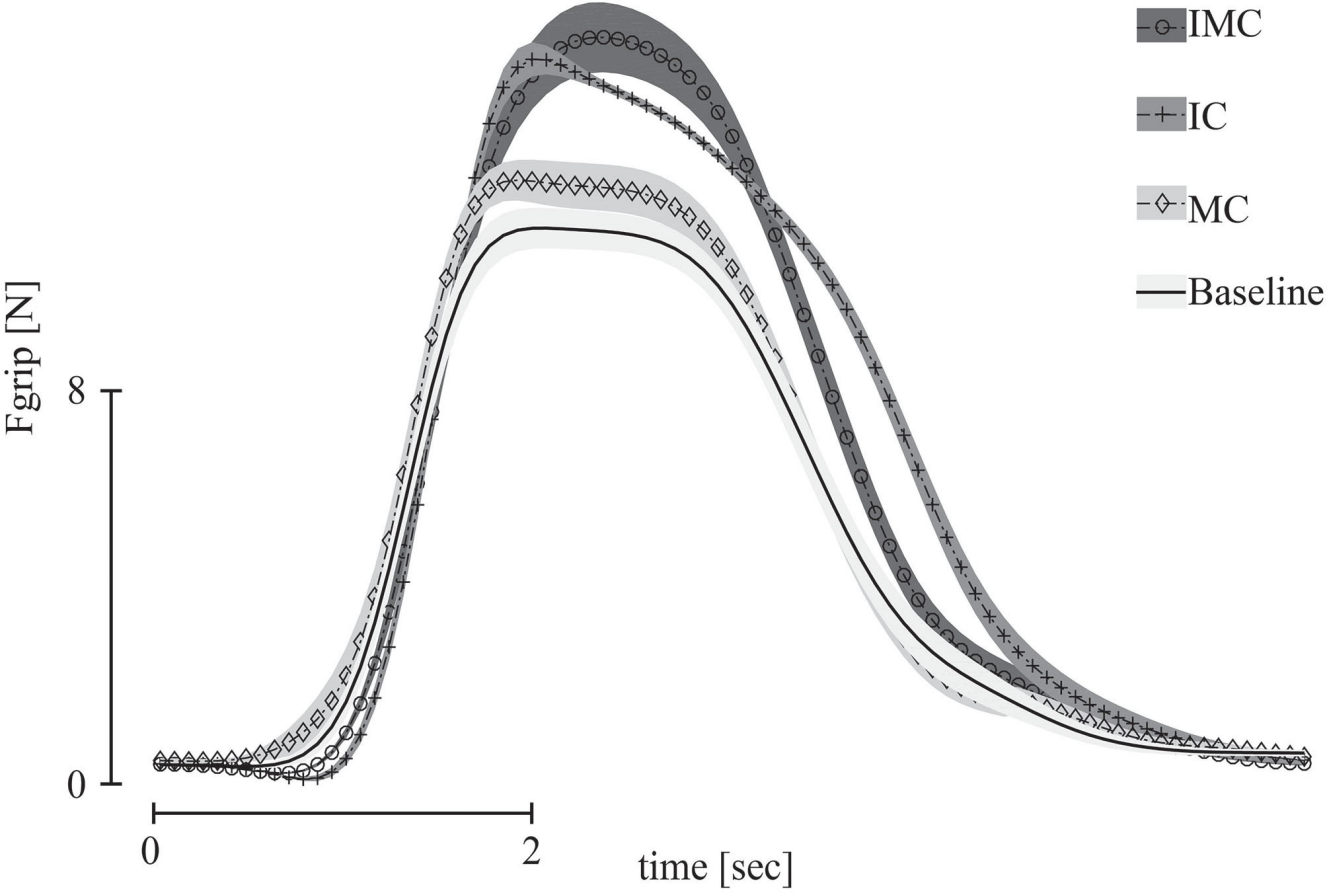
Grip force profiles in all glove conditions in three-digit grasp for all participants.

Before analyzing the forces exerted by each finger during three-digit grasping, we evaluated the overall grip force of the grasp and we report on the peak of the grip force. Participants produced the lowest peak grip force in the baseline condition (no glove), whereas they exerted the largest peak grip force when covering both fingers opposing the thumb, the index, and middle fingers. For the conditions with only one finger covered (index or middle finger), the result for the peak grip force was in-between: the peak grip force was slightly larger than baseline when covering the middle finger. When covering the index finger, the peak grip force was slightly higher compared with covering the middle finger but lower than the peak grip force with both fingers covered (Figure 5).

**Figure 6:**
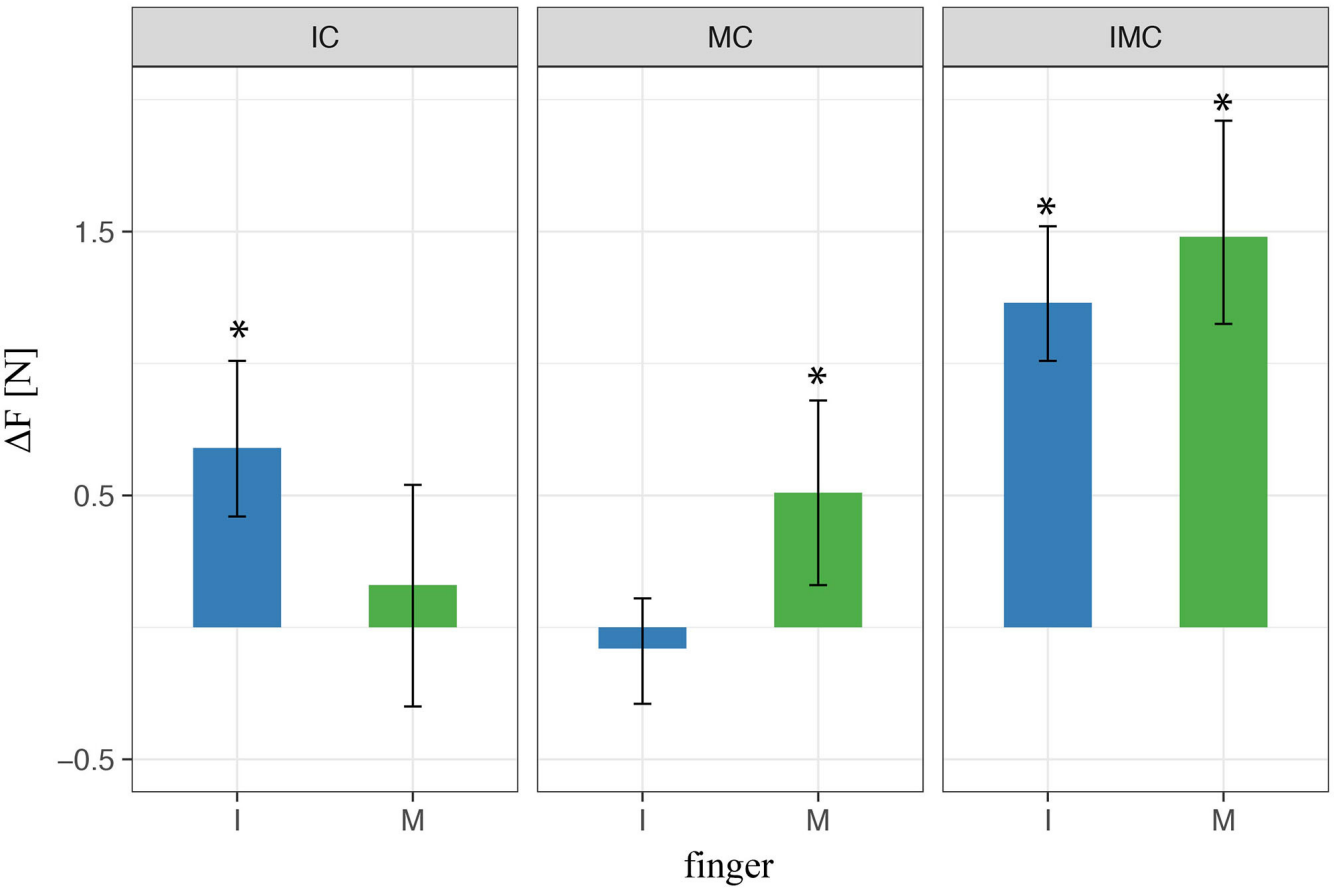
LMM results of digit normal forces in three-digit grasp. ^*^ indicate statistical significance determined by LMM analysis.

In order to get better insight on force control within single finger, we analyze the force contribution of each finger (mean values within transporting phase between *t*_0_ and *t*_1_) to the overall grip force. Figure 6 shows the force difference between the baseline condition (no glove) and the condition when at least one finger is covered (i.e., index or/and middle). When one of the two fingers opposing the thumb was covered during the three-digit grasps, the results using LMM revealed a significantly higher mean finger force compared with baseline for the covered finger (i.e., for the index in index-covered condition and the middle finger in the middle-covered condition). In contrast, there was nonsignificant effect of the noncovered finger (i.e., of the middle finger in index-covered condition or the index finger in the middle-covered condition) compared with baseline regarding the produced normal force. In the condition with both digits covered, we found a significant increase in finger force for both digits relative to baseline. These results indicate that the tactile perturbation significantly increased the produced finger force selectively for the digits covered.

**Figure 7:**
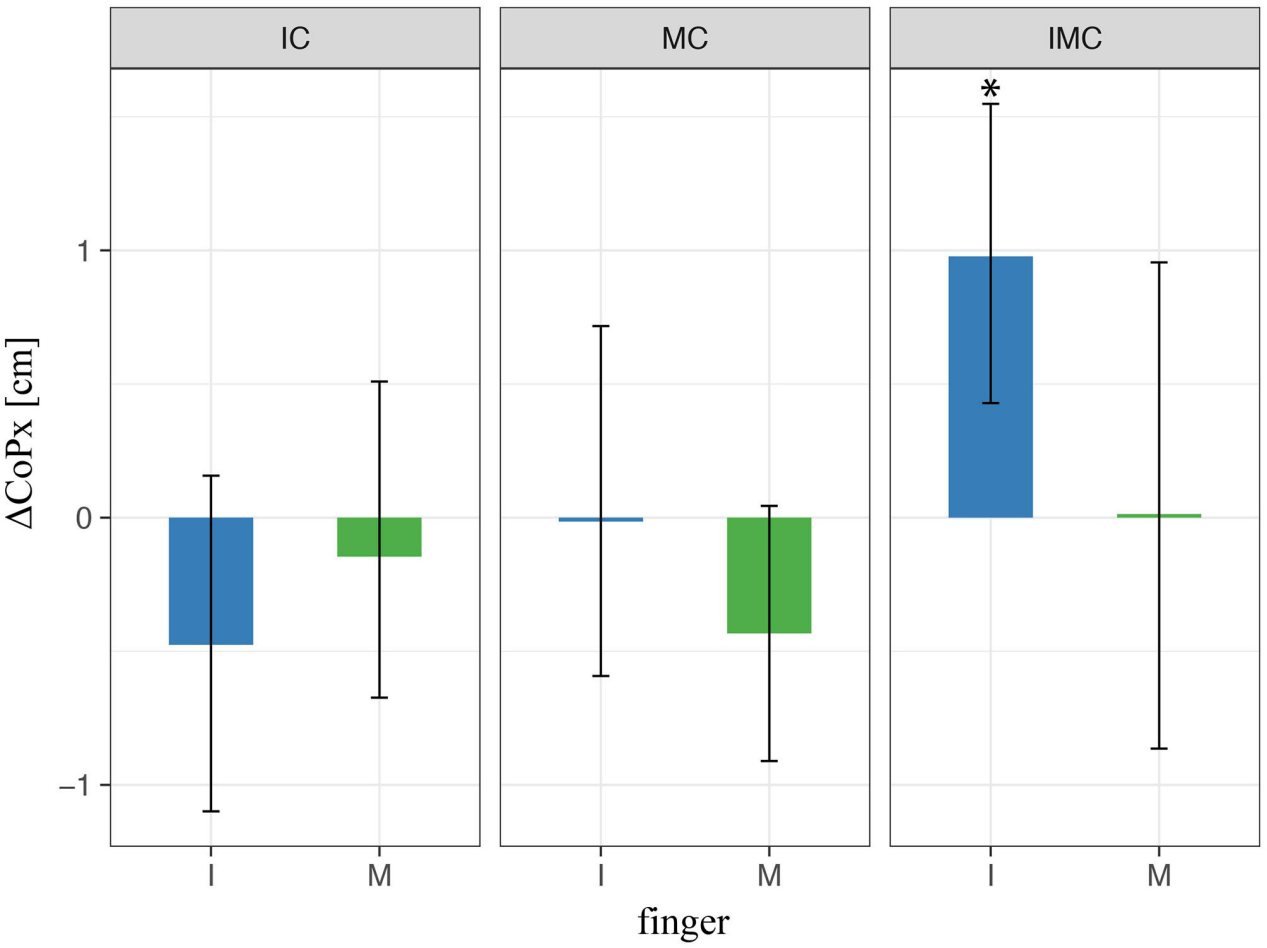
LMM results of digit initial horizontal placements (CoPs) in three-digit grasp. ^*^ indicate statistical significance determined by LMM analysis.

Next, we explored the horizontal placement of the fingers on the TACO. To this end, we compared the fingers' horizontal positions relative to the thumb for the glove and the baseline conditions. Figure 7 shows the difference in CoP_*x*_ between the baseline condition (no glove) and the condition when at least one finger is covered (i.e., index and/or middle). We found a significant variation in digit placement in the glove condition compared with baseline only for the index finger when both index and middle fingers were covered. The rest of the conditions were not significant (Figure 7); thus, these results indicate that the variation in digit placements cannot fully account for the effects found for the digit normal forces. Finally, we have to mention that there was no significant effect (*p* > 0.05) relative to the baseline condition in any of the conditions with different fingers covered on the vertical placement of the fingers (CoP_*y*_). Therefore, CoP_*y*_ was not included as a predictor in **Eqs. (4–6)**.

**Figure 8:**
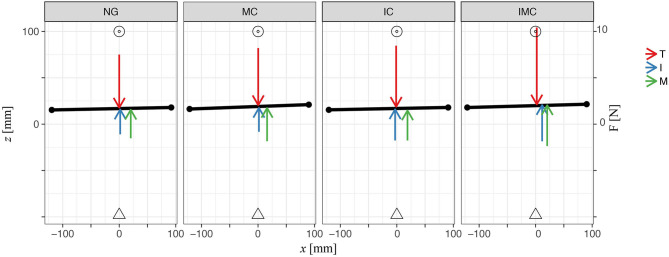
Plot illustrates average values across participants of digit normal forces, horizontal initial placements on the TACO, and the TACO yaw angle four digits within all glove conditions in three-digit grasp. Triangle represents the TACO start location, and circles represent the TACO target location.

Next, we analyze participants' grasp performance by investigating the yaw and roll angles of the TACO during the transport phase evaluating the mean (quantified within transport time window) orientation of the TACO. Participants kept the TACO relatively straight during transport and there was no significant difference of the mean orientation between the baseline and any of the glove conditions on the TACO orientations [likelihood ratio test; *α*-IC: $\chi_{\left(1\right)}^{2}$ = 0.61, *p* = 0.44; MC: $\chi_{\left(1\right)}^{2}$ = 1.48, *p* = 0.22; IMC: $\chi_{\left(1\right)}^{2}$ = 0.22, *p* = 0.64, *γ*-IC: $\chi_{\left(1\right)}^{2}$ = 0.98, *p* = 0.32; MC: $\chi_{\left(1\right)}^{2}$ = 3.16, *p* = 0.08; IMC: $\chi_{\left(1\right)}^{2}$ = 0.22, *p* = 0.64]. Figure 8 shows a top view of the experimental scene indicating averages across participants for the mean orientation of the TACO in yaw together with the mean normal force produced by each digit. This figure provides a schematic overview on the distribution of the mean normal forces across the fingers together with the horizontal digit placement on the TACO.

### Four-Digit Grasp

**Figure 9:**
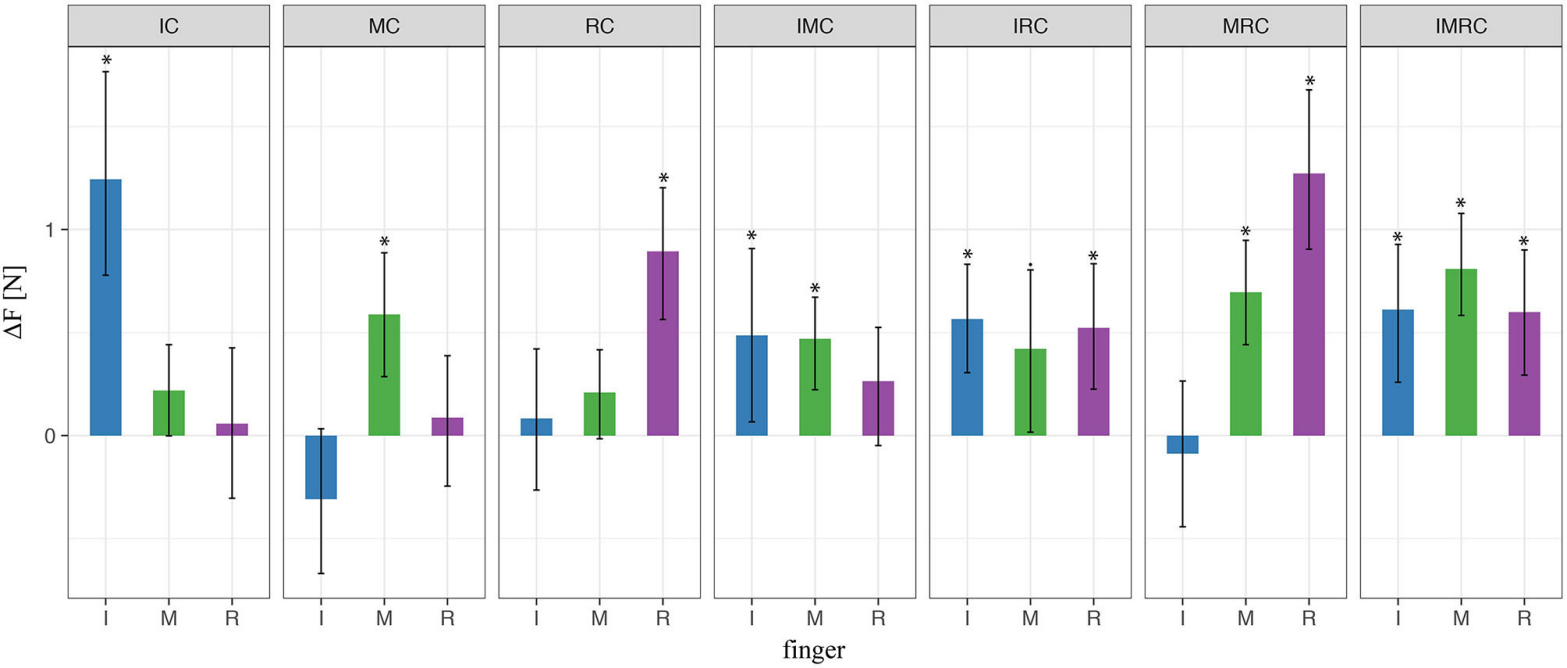
LMM results of digit normal forces in four-digit grasp. ^*^ indicate statistical significance determined by LMM analysis.

In this experiment, participants were grasping the TACO with four fingers—three fingers opposing the thumb—and either none, one, two, or three of the fingers opposing the thumb could be covered by the rubber thimble. Similar to the previous experiment, the present results of all conditions revealed a significant increase in the mean force of the finger or the fingers covered with the rubber thimble, while the other noncovered finger(s) did not differ significantly from baseline. This is shown in Figure 9 which for all fingers opposing the thumb depicts the difference in peak force averaged across participants between the experimental condition with at least one finger covered and the baseline with no finger covered. These results indicate that the tactile perturbation introduced significantly increased the digit force selectively for the covered finger also during four-digit grasps, similar as it did for three-digit grasps.

**Figure 10:**
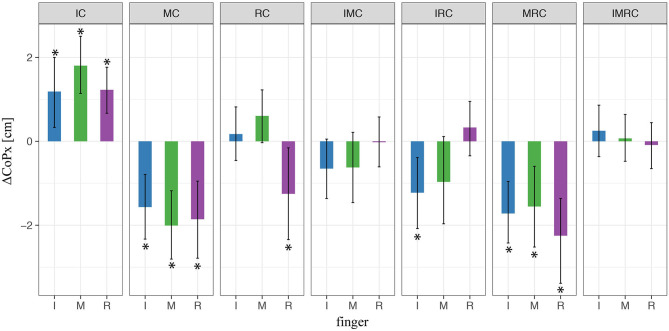
LMM results of digit initial horizontal placements (CoPs) in four-digit grasp. ^*^ indicate statistical significance determined by LMM analysis.

**Figure 11:**
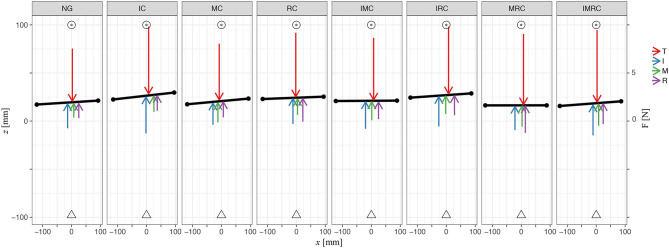
Plot illustrates average values across participants of digit normal forces, horizontal initial placements on the TACO, and the TACO yaw angle four digits within all glove conditions in four-digit grasp. Triangle represents the TACO start location, and circles represent the TACO target location.

Next, we explored how the fingers' horizontal positions varied relative to the thumb *Δ*CoP_*x*_ in the experimental conditions with tactile perturbation when compared with baseline (*Δ*CoP_*x*,exp_ – *Δ*CoP_*x*,baseline_, Figure 10). The patterns of observed changes in the placement of the fingers on the object seem meaningful for achieving a stable grasp with the altered digit forces reported above, which can be best seen in Figure 11. Figure 11 (top view) shows the average values across participants of the (mean values within transporting phase between *t*_0_ and *t*_1_) orientation of the TACO object (yaw angle) and the exerted digit normal forces for the fingers placed on the object. Thus, this figure provides an overview on the patterns of finger placement and forces when the tactile input was manipulated in the different conditions. For example, Figures 10, 11 show that we observed significant changes for the placement of all fingers to the right (top view) when the index finger was covered. The changes in finger placement counteract the higher digit force of the index finger that would otherwise induce an increased torque. Similarly, when the middle finger was covered which in baseline is typically placed left of the thumb, the placement of all fingers was slightly shifted to the left to counteract the higher forces of the middle finger when covered thus to not induce an unnecessary torque. The other patterns of changes in digit placement when perturbing the tactile input are also consistent with the notion of torque reduction due to an increased force in the covered digits. That is participants know or have learned over trials how to best reposition their fingers on the object to reduce torque when the grip forces are increased due to the tactile manipulation.

In order to investigate whether the torque due to increased digit forces was completely compensated by the adjustment of the placement of the fingers on the object, we analyzed the orientation (mean values of yaw and roll within transporting phase between *t*_0_ and *t*_1_) of the TACO object during the movement.

In general, participants kept the TACO object pretty straight in the glove condition compared with the baseline such that there was no significant effect of glove condition on its orientation [likelihood ratio test; *α*-IC: $\chi_{\left(1\right)}^{2}\;=$ 3.05, *p* = 0.08; MC: $\chi_{\left(1\right)}^{2}$ = 0.64, *p* = 0.43; RC: $\chi_{\left(1\right)}^{2}$ = 3.63, *p* = 0.06; IMC $\chi_{\left(1\right)}^{2}$ = 0.14, *p* = 0.71; IRC: $\chi_{\left(1\right)}^{2}$ = 0.05, *p* = 0.83; MRC: $\chi_{\left(1\right)}^{2}$ = 2.23, *p*= 0.14; IMRC: $\chi_{\left(1\right)}^{2}$ = 0.73, *p* = 0.39, γ-IC: $\chi_{\left(1\right)}^{2}$ = 0.36, *p* = 0.55; MC: $\chi_{\left(1\right)}^{2}$ = 0.37, *p* = 0.54; RC: $\chi_{\left(1\right)}^{2}$ = 0.27, *p* = 0.61; IMC $\chi_{\left(1\right)}^{2}$ = 0.36, *p* = 0.55; IRC: $\chi_{\left(1\right)}^{2}$ = 0.35, *p* = 0.55; MRC: $\chi_{\left(1\right)}^{2}$ = 2.35, *p* = 0.11; IMRC: $\chi_{\left(1\right)}^{2}$ = 0.74, *p* = 0.39]. There was also no difference in the orientation of the TACO between the experimental and the baseline condition, indicating that the torque due to increased digit forces was counteracted well by the repositioning of the fingers. Figure 11 shows a top view of the experimental scene indicating averages across participants for the mean orientation of the TACO in yaw together with the mean normal force produced by each digit. This figure provides a schematic overview on the distribution of the mean normal forces across the fingers together with the horizontal digit placement on the TACO.

## Discussion

In this work, we revisited the impact of tactile input on the modulation of the grip force during unconstrained grasping. All previous studies were conducted using constrained grasping. As in our previous studies, we found that digit normal forces are crucially altered by digit placements. In the present study, participants had to lift, transport, and replace the TACO with either three- and four-digit unconstrained grasp. Fingertips were covered with a rubber thimble depending on the experimental conditions. Overall, we recorded digit normal force changed locally when perturbing at least one finger opposing the thumb in three- and four-digit unconstrained grasp. Moreover, there was no systematic effect of glove condition on digit placements and the TACO tilt. Our results confirm and extend previous studies on local digit friction perturbation in two-, three-, and five-digit constrained grasp.

### Overall Grip Force Adjustment

Grip force was larger when fingers were covered with the rubber thimble compared with the baseline condition. This motor behavior is in accordance with previous studies that perturbed tactile input by changing the surface texture (Johansson and Westling, 1984; Burstedt et al., 1999; Aoki et al., 2007) or by asking participants to wear a glove (Kinoshita, 1999). Specifically, it has been shown that grip force increased with decrease of object friction between digit and object in order to maintain stability of an object (Johansson and Westling, 1984). The latter results were also confirmed by a study of Cadoret and Smith (1996) which showed that participants relied on the friction between digit and the grasped object to optimally modulate the grip force. The normal forces of the thumb and the opposing finger varied in a synergistic manner to increase the safety margin during the transport phase of the grasped object.

Instead, a reduction in the grip force was reported when perturbing tactile input using local anesthesia (Carteron et al., 2016). Specifically, Carteron et al. (2016) recorded a significant drop in the grip force when applying anesthesia at least in one of the digits involved in the grasp. Covering the fingers with the glove did not completely abolish cutaneous information at fingertips which is mainly responsible for generating an adequate safety margin at the individual fingers (Augurelle et al., 2003). However, this dual effect of covering the skin would not change the interpretation of the main finding, that is, the system responds to this perturbation at the level of individual digit.

### Digit Normal Force Control and Synergies

At the individual digit, when covering at least one finger only, the digit normal force changed in both covered and noncovered finger(s), but it reached significance level only for the covered finger(s). The latter result indicates that digit normal force changed locally at the fingers opposing the thumb. Similar observation was found in two-digit (Edin et al., 1992) and three-digit constrained grasp (Burstedt et al., 1999). To achieve a stable grasp in our experimental paradigm, thumb normal force should be equal to the sum of normal forces of the opposing fingers (see section “Methods”). Due to this mechanical constraint, the covered opposing finger(s) regulated locally the normal force that consequently altered the thumb. This latter was confirmed by looking at the TACO yaw and roll rotation angles that did not significantly varied with the force sharing that changed across conditions. Therefore, our results suggest that digit normal forces were adjusted locally for the covered and noncovered fingers whereas force increase at the covered finger altered the thumb normal force (opposing finger) as consequence of the task mechanical constraints. This latter result was also observed and reported in Carteron et al. (2016) where they used local anesthesia to disable cutaneous information that decrease the overall grip force. This latter work reported no synergistic motor behavior between fingers at the level of normal forces opposing the thumb which somehow underscore our obtained results to some extent. However, Aoki et al. (2007) examined the role of tactile input on the grip force modulation during five-digit constrained grasp and observed that varying friction condition between digits and the grasped object elicit force adjustment at both the stimulated and unstimulated digits.

The force sharing distribution among digits is also unconstrained due to the redundancy in degrees of freedom in terms of digit locations and forces (Friedman and Flash, 2007; Naceri et al., 2014, 2017). Indeed, distribution of digit normal forces was quite different between participants and they could still achieve a stable grasp. Considering this variability between participants, the local effect of the glove at the stimulated finger(s) persisted in all conditions. Previous studies on precision grip control showed that the magnitude of digit normal forces was regulated locally, within the perturbed digits, in two- and three-digit constrained grasp (Edin et al., 1992; Burstedt et al., 1999). The latter studies suggested that digit normal forces changes in parallel at all digits engaged in the grasping and manipulation tasks. It has been suggested that fast afferent fibers at fingertips (FA I) plays a role in triggering the controller of digit normal force due changes in the friction conditions between fingertip and the object signals in tactile afferents from fingers (Johansson and Westling, 1987). The local changes at frictional condition at fingertips play a role at triggering the digit force controller individually, which consequently scaled the overall grip force. Moreover, this overall change at the grip force due to the local frictional changes at each digit indicates that participants integrated the frictional condition at the covered fingers used in the grasping task. Especially, it has been shown that participants use frictional information from previous trials to scale the force output in anticipatory fashion to the frictional conditions at fingertips (Edin et al., 1992; Cole and Johansson, 1993; Johansson and Flanagan, 2009).

The friction of dry skin is characterized by relatively low and pressure-independent friction coefficients similar to the case of dry friction of rough solids whereas moist or wet skin shows high friction coefficients that strongly increase with decreasing contact pressure and are determined by the shear properties of wet skin (Derler and Gerhardt, 2012). The frictional behavior at the single digit tends to be extremely complex, and it might require complex models to understand such a behavior (Adams et al., 2013). This complexity is mainly due to several factors such as the unusual contact mechanics associated with fingerprint ridges and the relatively large number of sweat glands under these ridges (Adams et al., 2013). In the dry state, a finger pad has a coefficient of friction that is comparable with glassy polymers. However, sustained sliding on a smooth impermeable countersurface triggers the process of the secretion of moisture from the sweat glands causing the coefficient of friction to increase by about an order of magnitude to values comparable with elastomers, which can also exhibit contact areas close to the nominal values due to the deformability of the surface asperities. Covering the fingers with a rubber thimble might have triggered the process of the secretion of moisture from the sweat glands leading to the increase of skin friction of the covered finger which consequently caused the local adaptation of the normal force to friction change between fingertips and the grasped object.

Previous studies on prehension synergies reported the exitance of trade-offs between synergies at the two assumed hierarchical levels: thumb-opposing fingers and between opposing fingers (Gorniak et al., 2007, 2009; Sun et al., 2011; Wu et al., 2012). These studies evaluated the motor synergy at those levels using method of computation of synergy index. For instance, high variance of both thumb and opposing finger forces for the same performance variables leads to an increase of synergy index, whereas the same force variance at the individual fingers leads to a decrease of synergy index. Previous studies made by Gorniak et al. (2009) and Wu et al. (2012) did not observe any motor synergy when evaluating the synergy index. In our study, no clear motor synergy coordination was recorded between fingers (opposing the thumb) when covering at least one finger with a rubber thimble. The latter might be explained by a decrease of synergy index at the individual finger level as found in previous studies.

### Variability in Digit Placements

Participants tend to largely variate their digit placements when allowing them to freely choose the placement of their digits (Friedman and Flash, 2007; Fu et al., 2010; Naceri et al., 2017). Moreover, trial-to-trial variability in digit horizontal locations alters the modulation of the grip force (Naceri et al., 2014) and load force (Burstedt et al., 1999; Fu et al., 2010). In our experimental task, participants could freely choose their digit placements on the object leading to infinite combinations of digit position forces in order to achieve a stable grasp. Based on these observations, we included the normalized digit horizontal placements to thumb in our fitting model of digit normal forces in order to reduce CoPs' noise effect.

In this experiment, digit horizontal initial placements significantly varied and altered the digit normal forces. These changes in initial digit horizontal locations reflect trial-to-trial variability and also condition-to-condition variability and not within trial variability since participants did not regrasp the TACO during the holding phase. In other words, participants did not control systematically digit positions in order to adjust the grip force, despite the effect of digit positions on the grip force. This latter effect was explained by idiosyncratic grasping strategies (Naceri et al., 2017). In spite of the variability in digit horizontal placements, digit normal forces was significantly regulated at the covered finger(s).

The TACO's rotation angles (roll and yaw) and digit initial placements were not affected by the glove, as shown in the result section. This latter agrees with what is found in Burstedt et al. (1999), where also the manipulandum tilt and digit locations were not affected by the changing friction between fingertips and the grasped object. In contrast, local anesthesia at the individual digit altered the object tilt (Carteron et al., 2016). Indeed, using gloves or a surface texture for instance to change local friction condition at fingertip contacts does not induce motor deficit as does local anesthesia. Importantly, the recorded normal force was affected by either above methods used to perturb the tactile input at fingertips.

## Conclusion

In summary, our results suggest that perturbing tactile gloves increased the overall grip force in order to provide and ensure a stable grasp, despite that the produced force is higher than the one required for the task. Digit normal forces were adjusted locally between fingers opposing the thumb when covered using the glove. The force increase adjustment at the covered finger(s) altered the thumb normal force since this latter digit mechanically contributes 50% to the overall grip force. There were no systematic effects across participants by glove conditions on the digit initial placements and TACO rotation angles. This latter effect is due to the fact these variables could vary between participants as well as between trials within participants.

## Data Availability Statement

The raw data supporting the conclusions of this article will be made available by the authors, without undue reservation, to any qualified researcher.

## Ethics Statement

The studies involving human participants were reviewed and approved by Ethics committee of Bielefeld University. The patients/participants provided their written informed consent to participate in this study.

## Author Contributions

AN and ME conceived and designed the study and coordinated the scientific and technical aspects of this work. YG conducted the experiments and data processing. AN and YG analyzed, processed data, and prepared the figures. AN, YG, AM, and ME wrote the manuscript. AN, AM and ME supervised the study. All authors contributed to the article and approved the submitted version.

## Conflict of Interest

The authors declare that the research was conducted in the absence of any commercial or financial relationships that could be construed as a potential conflict of interest.
